# Associations between maternal thyroid hormones and neonatal outcomes during pregnancy

**DOI:** 10.3389/fendo.2026.1850925

**Published:** 2026-06-08

**Authors:** Lin Tao, Yi Zhang, Yuan Zhongzhou, Shen Xubo

**Affiliations:** 1 School of Public Health, Zunyi Medical University, Zunyi, Guizhou, China; 2 The People’s Hospital of Qian Nan Prefecture, Qian Nan, Guizhou, China; 3 Centre for Disease Control and Prevention, Guiyang, Guizhou, China

**Keywords:** endocrine, gender differences, maternal thyroid hormones, neonatal birth outcomes, pregnancy

## Abstract

**Introduction:**

This study aims to explore the gestational dynamics of maternal thyroid hormones and their associations with neonatal birth outcomes (birth weight, length, and parity), including 2,884 pregnant women (287 in early, 807 in mid, and 1,487 in late pregnancy).

**Methods:**

Using univariate ANOVA to assess trimester-specific thyroid hormone differences, linear mixed-effects models to examine associations with neonatal outcomes, restricted cubic spline (RCS) plots to evaluate dose-response relationships, and Bayesian kernel machine regression (BKMR) to analyze combined effects of thyroid hormone mixtures.

**Results:**

We found that maternal Thyroid Stimulating Hormone (TSH), Total Triiodothyronine (TT3), Free Triiodothyronine (FT3), and Free Thyroxine (FT4) levels changed significantly with gestation (TSH increasing gradually, TT3 peaking in mid-pregnancy, and FT3/FT4 declining continuously), Weak correlations were observed between maternal thyroid hormones and neonatal outcomes, with mixed linear regression revealing trimester- and sex-specific associations of TT4, TT3, FT3, and FT4 with birth weight, length, and parity. RCS analysis further showed dose-response relationships with pregnancy-stage and sex-specific patterns, while BKMR indicated weak positive correlations of thyroid hormone mixtures with neonatal outcomes in early/mid-pregnancy and significant positive correlations in late pregnancy. The relative contribution of each thyroid hormone to neonatal outcomes was also quantified.

**Conclusion:**

Collectively, our findings highlight trimester-specific thyroid hormone dynamics, complex dose-response relationships with neonatal outcomes, and stage/sex-dependent associations, underscoring the need for gestational thyroid function monitoring to inform maternal–infant health strategies.

## Introduction

Thyroid hormones, synthesized and secreted by thyroid follicular cells, are a class of key regulators. Clinically, their levels are commonly assessed through measurements of thyroid-stimulating hormone (TSH), total thyroxine (TT4), free thyroxine (FT4), total triiodothyronine (TT3), and free triiodothyronine (FT3). Notably, only the free fractions of these hormones exhibit biological activity (1). The actions of thyroid hormones are mediated through multiple mechanisms. First, they govern metabolic homeostasis by promoting oxidative-reductive reactions, thereby increasing basal metabolic rate and thermogenesis, and orchestrating the metabolism of carbohydrates, fats, and proteins. This metabolic regulation is essential for satisfying the energy demands of both the mother and fetus during pregnancy. Insufficient thyroid hormone secretion reduces maternal metabolic rate, which can subsequently impede fetal nutrient supply and compromise growth and development (2). Second, thyroid hormones are indispensable for the development of the fetal nervous and skeletal systems. In the early stages of pregnancy, the fetal thyroid is immature, and its development relies entirely on maternal-derived thyroid hormones. These hormones drive critical processes such as neuronal proliferation, migration, differentiation, and myelination, all of which are fundamental for normal brain development. Maternal thyroid hormone deficiency is associated with fetal short stature (3). Finally, thyroid hormones contribute to maintaining cardiovascular homeostasis during pregnancy by enhancing myocardial contractility, increasing heart rate, and augmenting cardiac output, thereby ensuring adequate blood circulation for both the mother and fetus (4).

However, studies simultaneously examining maternal thyroid hormone dynamics across all trimesters remain limited. In early pregnancy, human chorionic gonadotropin (hCG)—structurally similar to TSH—stimulates thyroid activity, causing mild elevations in TT4 and FT4. FT4 levels then gradually decline, remaining low until delivery. hCG suppresses TSH in early pregnancy, with levels gradually increasing in mid-to-late gestation while remaining relatively low (5). Notably, thyroid hormone reference ranges differ across gestational stages (6, 7).

Multiple studies have reported associations between maternal thyroid hormone levels and neonatal birth weight (21). For instance, early-pregnancy maternal thyroid-stimulating hormone (TSH) levels correlate negatively with newborn birth weight, while elevated free thyroxine (FT4) may also reduce birth weight (1, 8, 9*)*. Thyroid dysfunction states, including clinical or subclinical hypothyroidism, increase the risk of low birth weight (3). However, inconsistent findings exist: some studies report no significant associations (4, 10, 11), while others find positive correlations between late-pregnancy FT3 and birth weight (5). Such heterogeneity likely arises from differences in study populations (e.g., race, region, environment) and biases in study design or sample size. Here, we aim to clarify the causal links and mechanisms between gestational thyroid hormone dynamics and neonatal birth outcomes through systematic analysis of thyroid hormone trajectories combined with multidimensional exposure assessment.

## Methods

### Study population

The study population was drawn from the Zunyi birth cohort. Inclusion criteria for this study: age between 20–45 years, spontaneous conception, and singleton pregnancy. Exclusion criteria: severe chronic and infectious diseases (e.g., cancer, cardiovascular disease, chronic renal failure, and HIV infection). The study was approved by the Ethics Committee of Zunyi Medical College (No. [2019] H-005) and each pregnant woman voluntarily signed an informed consent form. A total of 2884 pregnant women were included in the study, divided into The First Trimester (≤ 12 weeks), The Second Trimester (13–27 weeks), and The Third Trimester (≥ 28 weeks) according to the number of weeks of pregnancy, with corresponding numbers of 287, 807, and 1487, respectively (**Supplementary Figure 1**).

### Thyroid hormones

In this study, thyroid hormone measurements were conducted at the hospital, which used electrochemiluminescence immunoassay (ECLIA) on an automated analyzer (Roche Cobas e601) to measure serum TSH, TT3, TT4, FT3, and FT4. However, the specific plan and quality control measures for thyroid hormone testing have not been documented.

### Neonatal outcomes

Neonatal birth outcomes comprised birth weight (g), birth length (cm), and birth parity (Birth weight divided by birth length to the power of 3: kg/m^3^). Data were retrieved from hospital records and validated by project team members through systematic review of institutional electronic health systems.

### Corrective factors

The correction factors came from a questionnaire survey during pregnancy. The correction factors in this study mainly include the mother’s age, education level, occupation, marital status, pre pregnancy body mass index, smoking history, spouse’s education level, spouse’s occupation, annual family income, and spouse’s smoking history.

### Statistical analysis

The Kolmogorov–Smirnov test was used to assess the normality of the data distribution. Continuous variables are presented as the mean ± standard deviation (SD), and categorical variables are presented as percentages. For non-normally distributed variables, median and interquartile range (IQR) are reported. As thyroid hormone concentrations exhibited right-skewed distributions, subsequent analyses were performed following natural logarithmic transformation. A linear mixed-effects regression model was employed to investigate associations between maternal thyroid hormone levels and neonatal birth outcomes. Restricted cubic spline (RCS) analysis was then applied to explore dose-response relationships. Additionally, Bayesian kernel machine regression (BKMR) was implemented to quantify the cumulative effects of maternal thyroid hormones on neonatal birth outcomes. Data entry and preliminary checks were conducted using SPSS 29.0 (IBM Corp.) and R version 3.4.0 (Comprehensive R Archive Network; http://cran.r-project.org). Statistical significance was defined as two-tailed *P* < 0.05.

## Results

### Baseline data

This study enrolled 2,884 mother-infant pairs, comprising 1,225 in the first trimester, 782 in the second trimester, and 877 in the third trimester. Participants were married women aged 20–35 years with at least a junior high school education. Approximately 40% had abnormal pre-pregnancy BMI (<18.5 kg/m² or >23.9 kg/m²), household annual income predominantly ranged between 100,000 and 200,000 yuan, and nearly all were non-smokers but were frequently exposed to secondhand smoke. Chi-square tests revealed significant intergroup differences in age, occupational history, pre-pregnancy BMI, spouse’s occupational history, household annual income, and delivery mode (**Table 1**).

**Table 1:** Baseline information about pregnant women.

Variable		The first trimester (N = 1225)	The second trimester (N = 782)	The third trimester (N = 877)	*X* ^2^	P
Age (years)	20–35 36–45	1172 (95.67%) 53 (4.33%)	736 (94.12%) 46 (5.88%)	812 (92.59%) 65 (6.41%)	9.147	**0.01**
Education	Low top High	33 (2.69%) 928 (75.76%) 264 (21.55%)	28 (3.58%) 613 (78.39%) 141 (18.03%)	20 (2.28%) 697 (79.48%) 160 (18.24%)	7.771	0.10
Employment status	Employed Unemployed	758 (61.88%) 467 (38.12%)	499 (63.81%) 283 (36.19%)	591 (67.39%) 286 (32.61%)	6.777	**0.034**
Marriage status	Married Unmarried	1057 (86.29%) 68 (13.71%)	735 (94.37%) 47 (5.63%)	833 (94.96%) 44 (5.06%)	0.789	0.674
Pre-pregnancy BMI (kg/m^2^)	<18.5 18.5–23.9 >23.9	159 (12.98%) 772 (63.02%) 294 (24.00%)	79 (10.10%) 474 (60.61%) 229 (29.29%)	115 (13.11%) 512 (58.38%) 250 (28.51%)	12.174	**0.016**
Spouse’s education	Low top High	15 (1.22%) 939 (76.65%) 271 (22.13%)	11 (1.41%) 624 (79.80%) 147 (18.79%)	9 (1.03%) 705 (80.39%) 163 (18.58%)	5.723	0.221
Spouse’s employment status	Employed Unemployed	1111 (90.69%) 114 (9.31%)	681 (87.08%) 101 (12.92%)	778 (88.71%) 99 (12.29%)	6.618	**0.037**
Annual household income (yuan)	<100000 100000–200000 >200000	177 (14.45%) 915 (74.69%) 133 (10.86%)	140 (17.90%) 574 (73.40%) 68 (8.70%)	149 (16.99%) 663 (75.60%) 65 (7.41%)	11.235	**0.024**
Active smoking	Yes No	24 (1.96%) 1201 (98.04%)	18 (2.30%) 764 (91.70%)	14 (1.60%) 863 (98.40%)	1.084	0.582
Spouse’s smoking	Yes No	505 (41.22%) 720 (58.78%)	345 (44.12%) 437 (55.88%)	345 (39.34%) 532 (60.66%)	3.930	0.140
Type of delivery	Natural birth Caesarean section	682 (55.67%) 543 (44.33%)	463 (59.21%) 319 (40.79%)	462 (52.68%) 415 (47.32%)	7.141	**0.028**
Sex of newborn	Male Female	622 (50.77%) 563 (59.23%)	439 (56.14%) 343 (43.86%)	477 (54.39%) 400 (45.61%)	2.575	0.376
Gestation week	NA	8.62 ± 2.55	18.85 ± 3.93	35.90 ± 3.90	NA	NA

Low: primary school education and below. top, junior high school education. High, college education and beyond.

The bold values represent P values less than 0.05.

### Maternal thyroid hormone levels during pregnancy and neonatal birth outcomes

This study categorized participants by gestational age into the first trimester, the second trimester (13–28 weeks), and the third trimester (>28 weeks). In the first trimester, maternal thyroid function parameters were as follows: TSH 1.42 (0.82, 2.24) mIU/L, TT3 1.42 (1.23, 1.63) nmol/L, TT4 10.22 (8.89, 11.92) nmol/, FT3 3.21 (2.99, 3.51) pmol/L, and FT4 1.27 (1.17, 1.40) pmol/L. Thyroid hormone levels across trimesters are detailed in **Table 2**. Analysis of variance revealed significant trimester-related differences in TSH, TT3, FT3, and FT4. Specifically, TSH increased progressively from the first trimester to the third trimester; TT3 rose from the first trimester to the second trimester before declining; FT3 and FT4 decreased continuously from the first trimester to the third trimester. By contrast, no significant gestational age-related differences were observed in neonatal birth weight, length, or body mass index (**Table 2**). Pearson correlation analysis showed weak associations between TSH and TT3, TT4, FT3, or FT4 (all r < 0.4), moderate correlations among TT3, TT4, FT3, and FT4 (0.44 < r < 0.6), and a strong positive correlation between FT4 and TT4 (r = 0.93). Birth weight, length, and parity showed moderate intercorrelations (0.78 < r < 0.84), whereas maternal thyroid hormones exhibited weak associations with these neonatal outcomes (r < 0.1) (**Supplementary Figure 2**).

**Table 2:** Thyroid hormone levels in maternal blood during pregnancy and neonatal birth outcomes.

	The first trimester (N = 1225) 50^th^ (25^th^, 75^th^)	The second trimester (N = 782) 50^th^ (25^th^, 75^th^)	The third trimester (N = 877) 50^th^ (25^th^, 75^th^)	*F*	*P*
TSH *(mIU/L)*	1.42 (0.82, 2.24)	1.88 (1.12, 2.78)	2.15(1.30, 3.53)	39.750	**<0.001**
TT3 *(nmol/L)*	1.42 (1.23, 1.63)	1.64 (1.42, 1.88)	1.41 (1.17, 1.66)	91.948	**<0.001**
TT4 *(nmol/L)*	10.22 (8.89, 11.92)	11.44 (9.90, 12.83)	10.24 (8.82, 11.94)	1.839	0.159
FT3 *(pmol/L)*	3.21(2.99, 3.51)	2.94 (2.68, 3.22)	2.61 (2.22, 3.04)	308.475	**<0.001**
FT4 *(pmol/L)*	1.27 (1.17, 1.40)	1.14 (1.03, 1.26)	1.07 (0.94, 1.23)	6.423	**0.002**
Birth weight *(g)*	3200 (2950, 3500)	3200 (2950, 3450)	3200 (2900, 3500)	1.031	0.357
Birth length *(cm)*	50.00 (50.00, 50.00)	50.00(50.00, 50.00)	50.00 (50.00, 50.00)	1.225	0.294
Birth parity *(kg/m^3^)*	25.60 (24.22, 27.14)	25.60 (24.22, 27.14)	25.60 (24.14, 27.13)	.376	0.687

The bold values represent P values less than 0.05.

### Association between maternal thyroid hormones and neonatal birth outcomes

**Figure 1:**
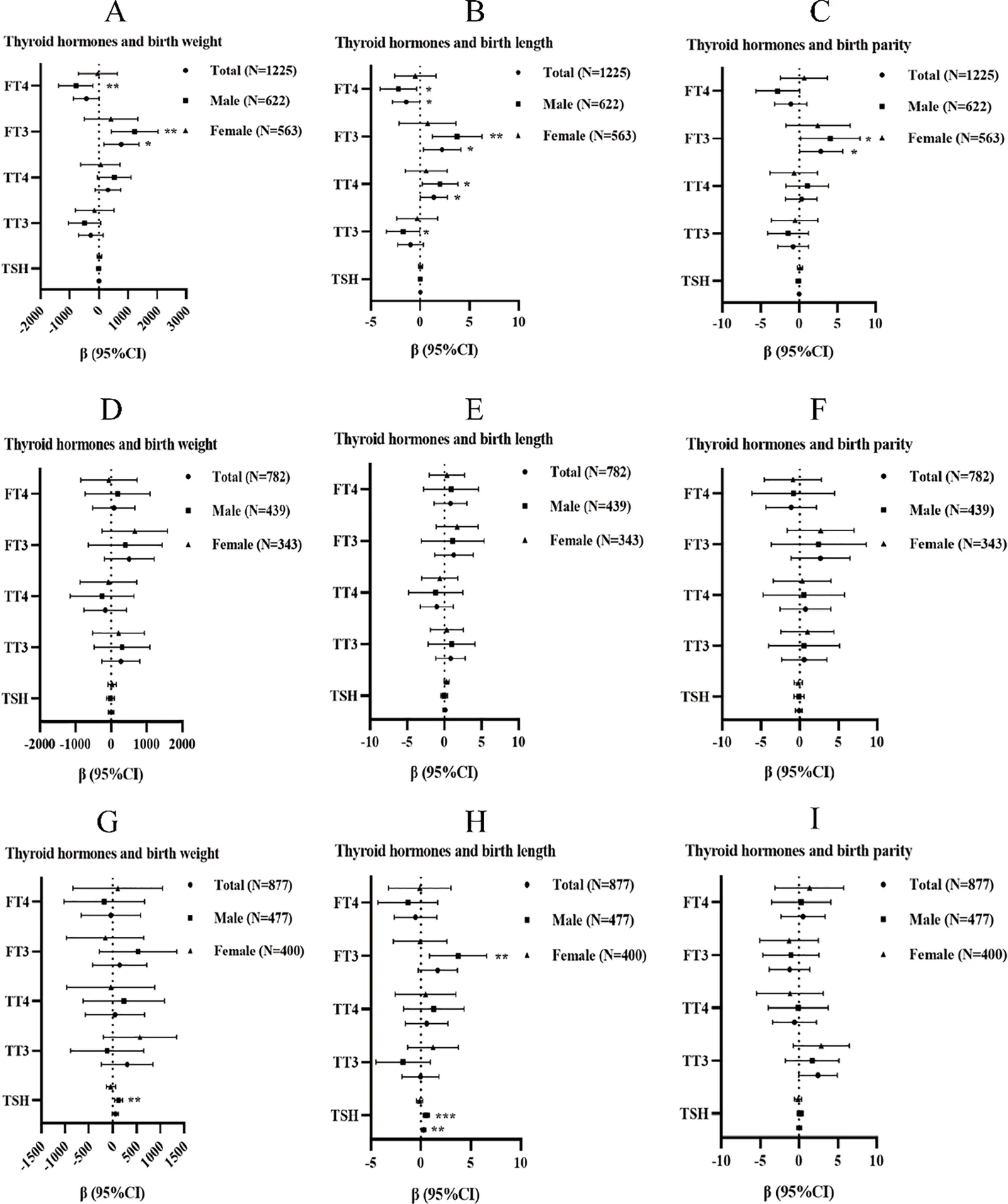
Association between maternal thyroid hormones and neonatal birth outcomes. **(A–C)** illustrate the association between maternal thyroid hormones and birth outcomes in the first trimester; **(D–F)** illustrate the association between maternal thyroid hormones and birth outcomes in the second trimester; **(G–I)** shows the association between maternal thyroid hormones and birth outcomes in the third trimester. Adjustment factors: maternal age, education, occupation, marital status, pre-pregnancy body mass index, smoking history, spouse’s education, spouse’s occupation, annual household income, and spouse’s smoking history.

A mixed linear regression model was used to evaluate associations between maternal thyroid hormones and neonatal birth outcomes. In the first trimester, before gender stratification, FT3 was positively associated with birth weight (β = 768.902, 95% CI: 167.145–1370.659); TT4, FT3, and FT4 were associated with birth length (β = 1.382, 95% CI: 0.013–2.752; β = 2.218, 95% CI: 0.337–4.100; β = -1.437, 95% CI: -2.814–0.061); and FT3 was associated with birth parity (β = 2.863, 95% CI: 0.029–5.696). After gender stratification, FT3 and FT4 exhibited divergent associations with male birth outcomes: FT3 was positively associated with male birth weight (β = 1,230.810, 95% CI: 426.357–2,035.263), while FT4 was negatively associated (β = -786.476, 95% CI: -1,375.876–197.077). For male birth length, TT3, TT4, FT3, and FT4 showed mixed associations (β = -1.728, 95% CI: -3.433–0.023; β = 2.019, 95% CI: 0.222–3.815; β = 3.753, 95% CI: 1.246–6.261; β = -2.221, 95% CI: -4.058–0.384), and FT3 was positively associated with male birth parity (β = 4.086, 95% CI: 0.197–7.974). No significant associations were observed between maternal thyroid hormones and female birth weight, length, or parity (**Figures 1A–C**). In the second trimester, no significant associations were detected between maternal thyroid hormones and neonatal birth outcomes in either gender (**Figures 1D–F**). In the third trimester, before gender stratification, TSH was positively associated with birth weight (β = 0.278, 95% CI: 0.060–0.496). After stratification, TSH was positively associated with male birth weight (β = 122.162, 95% CI: 37.592–206.732), and both TSH and FT3 were associated with male birth length (β = 0.543, 95% CI: 0.244–0.842; β = 3.724, 95% CI: 0.862–6.586). No significant associations were found for male birth parity or any outcomes in females (**Figures 1G–I**).

### Dose-response relationship between maternal thyroid hormones and neonatal birth outcomes

**Figure 2:**
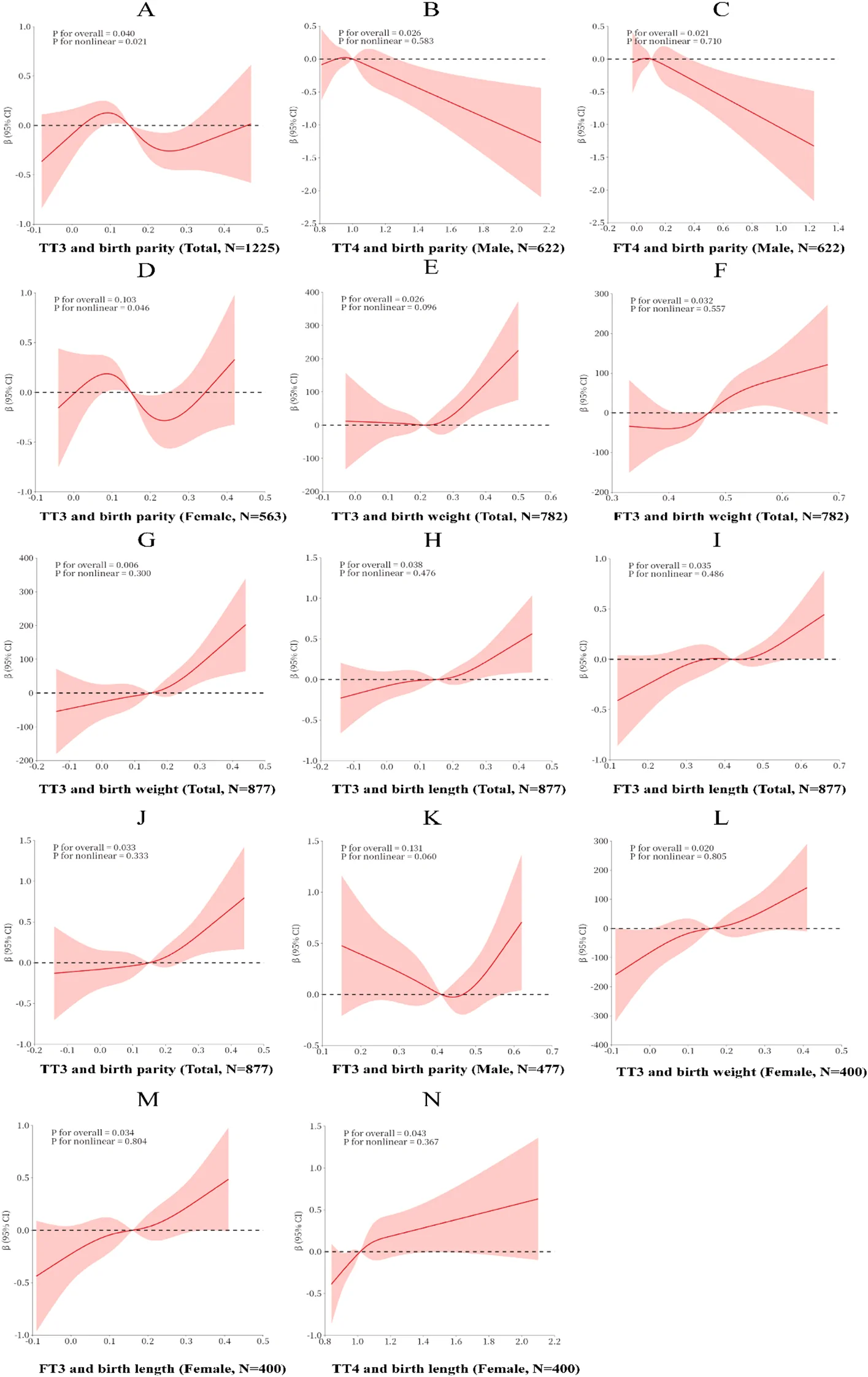
Dose-response relationship between maternal thyroid hormones and neonatal birth outcomes. **(A–D)** illustrate the non-linear relationship between maternal thyroid hormones and birth outcomes in the first trimester; **(E, F)** illustrate the non-linear relationship between maternal thyroid hormones and birth outcomes in the second trimester; **(G–N)** shows the non-linear relationship between maternal thyroid hormones and birth outcomes in the third trimester. Adjustment factors: maternal age, education, occupation, marital status, pre-pregnancy body mass index, smoking history, spouse’s education, spouse’s occupation, annual household income, and spouse’s smoking history.

In this study, restricted cubic spline (RCS) curves were used to explore non-linear associations between maternal thyroid hormones and neonatal birth outcomes. In the first trimester, before gender stratification, TT3 exhibited an inverted S-shaped correlation with birth parity (overall P = 0.040; non-linear P = 0.021). After stratification, TT4 and FT4 showed non-linear negative associations with male birth parity (P = 0.026 and 0.021, respectively), while TT3 displayed an inverted S-shaped relationship with female birth parity (non-linear P = 0.046). In the second trimester, unstratified analyses revealed non-linear positive associations of TT3 and FT3 with birth weight (P = 0.026 and 0.032, respectively), which were not evident after gender stratification. In the third trimester, unstratified RCS analysis showed non-linear positive associations of TT3 with birth weight, length, and parity (P = 0.006, 0.038, and 0.033, respectively), and FT3 with birth length (P = 0.035). After gender stratification, FT3 exhibited an inverted U-shaped correlation with male birth weight (non-linear P = 0.06), TT3 showed a non-linear positive association with female birth weight (P = 0.02), and FT3 and TT4 were non-linearly positively associated with female birth length (P = 0.034 and 0.043, respectively) (**Figure 2**).

### Overall association between maternal thyroid hormones and neonatal birth outcomes

**Figure 3:**
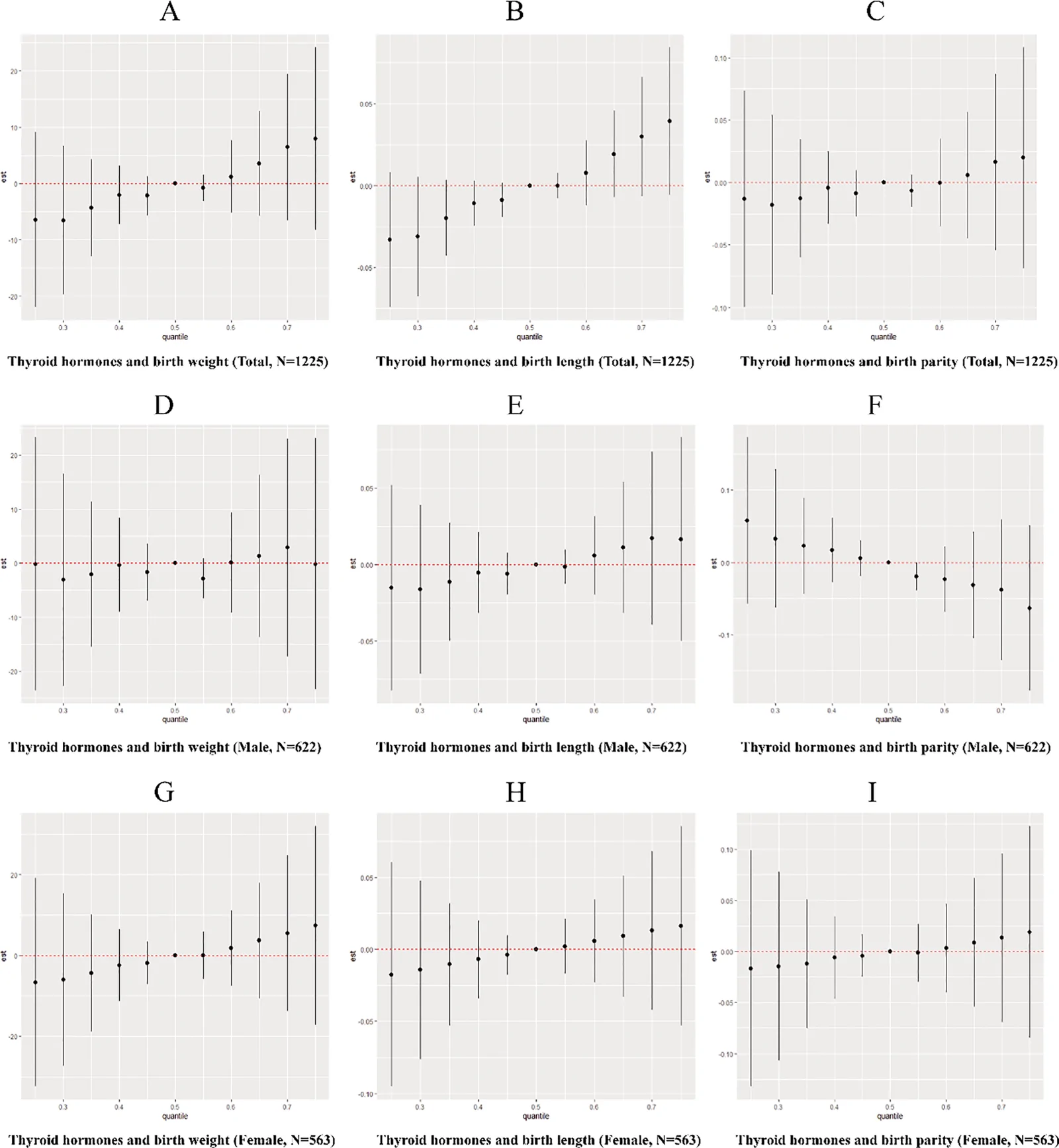
Comprehensive association between the first trimester thyroid hormone mixtures and neonatal birth outcomes. Adjustment factors: maternal age, education, occupation, marital status, pre-pregnancy body mass index, smoking history, spouse’s education, spouse’s occupation, annual household income, and spouse’s smoking history. **(A–C)** show the combined relationship between maternal thyroid hormones and overall neonatal birth outcomes; **(D–F)** show the combined relationship between maternal thyroid hormones and male newborn birth outcomes; **(G–I)** shows the combined relationship between maternal thyroid hormones and the birth outcomes of female newborns.

**Figure 4:**
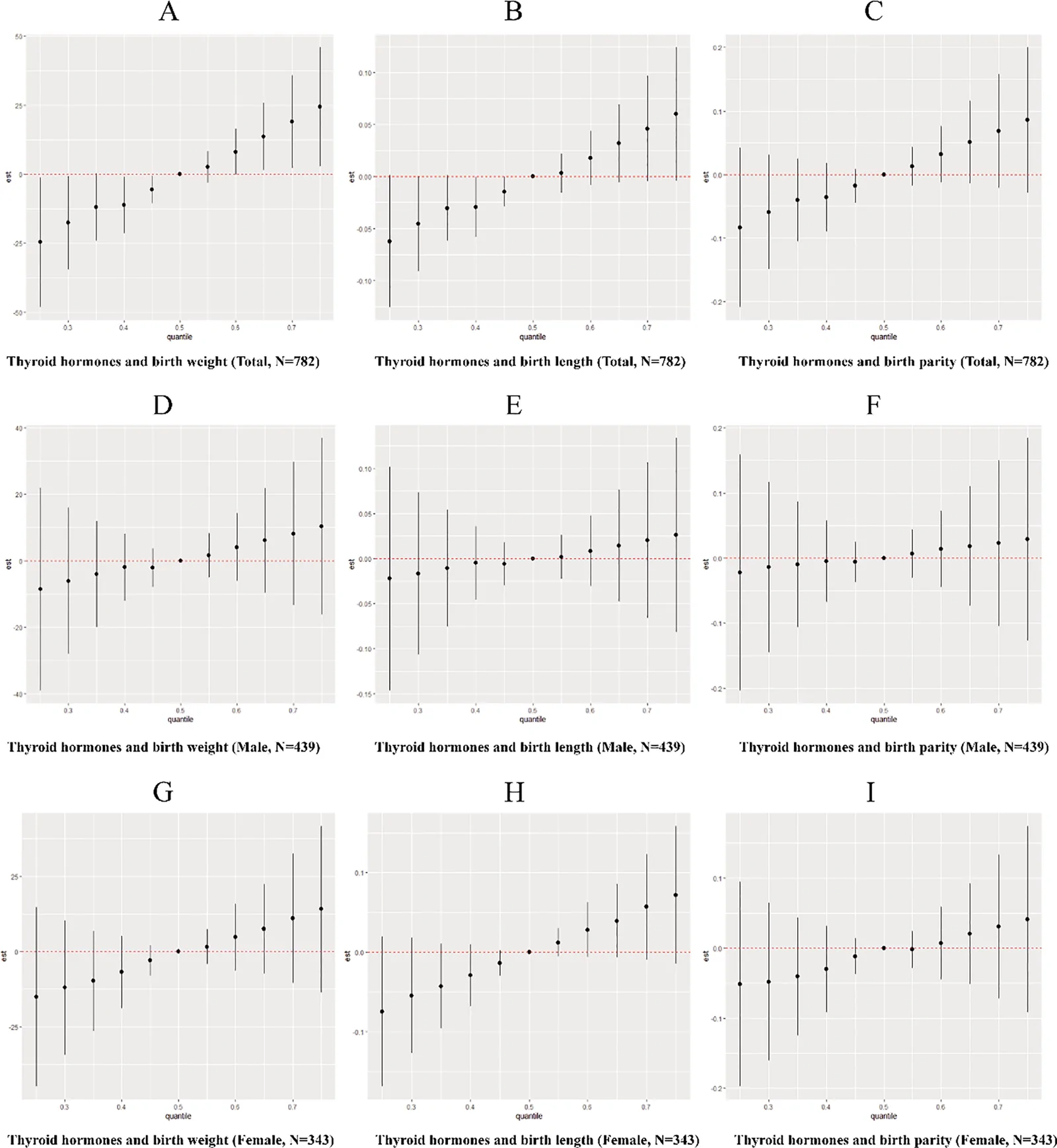
Comprehensive association between the second trimester thyroid hormone mixtures and neonatal birth outcomes. Adjustment factors: maternal age, education, occupation, marital status, pre-pregnancy body mass index, smoking history, spouse’s education, spouse’s occupation, annual household income, and spouse’s smoking history. **(A–C)** show the combined relationship between maternal thyroid hormones and overall neonatal birth outcomes; **(D–F)** show the combined relationship between maternal thyroid hormones and male newborn birth outcomes; **(G–I)** shows the combined relationship between maternal thyroid hormones and the birth outcomes of female newborns.

**Figure 5:**
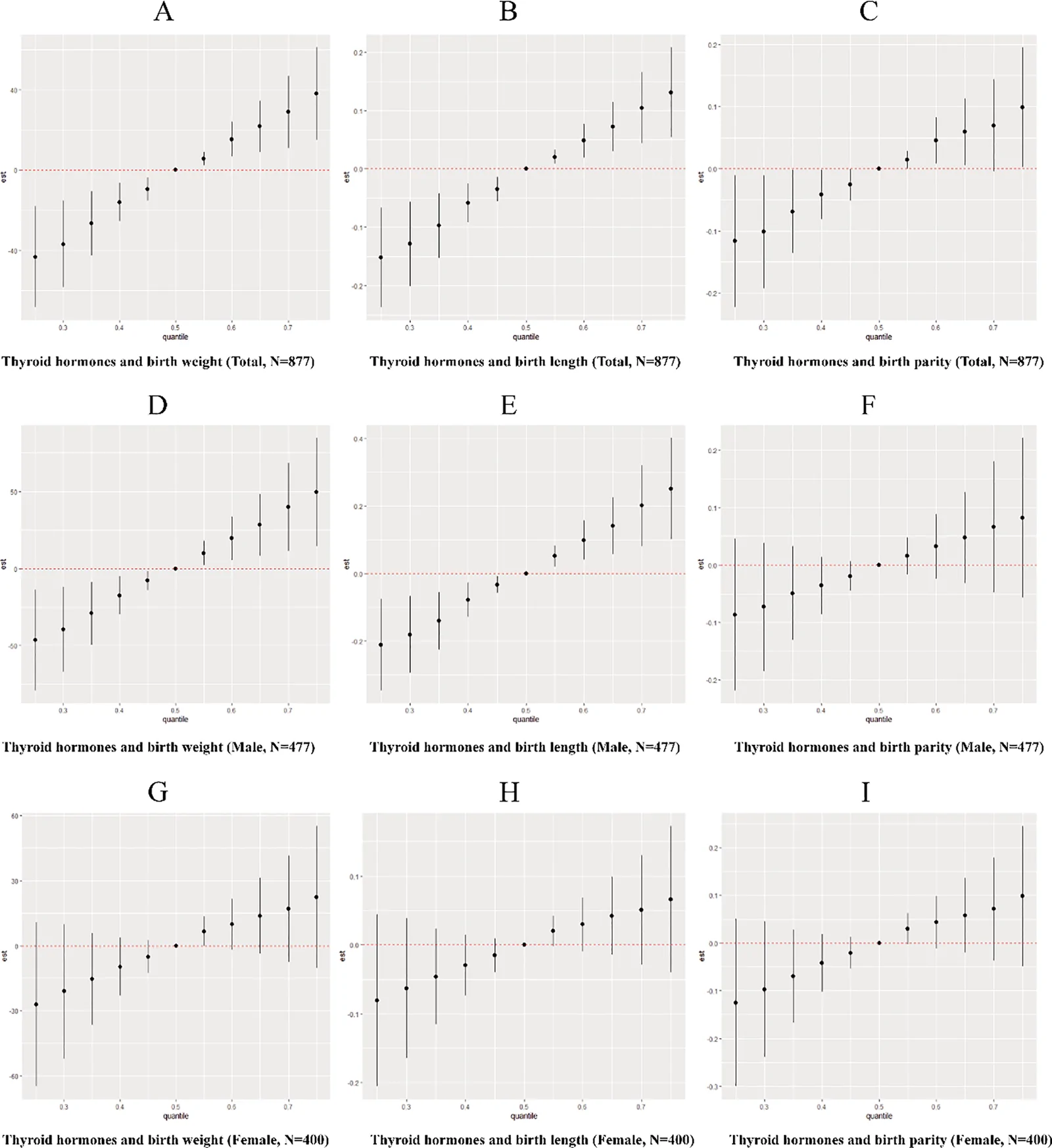
Comprehensive association between thyroid hormone mixtures in the third trimester and neonatal birth outcomes. Adjustment factors: maternal age, education, occupation, marital status, pre-pregnancy body mass index, smoking history, spouse’s education, spouse’s occupation, annual household income, and spouse’s smoking history. **(A–C)** show the combined relationship between maternal thyroid hormones and overall neonatal birth outcomes; **(D–F)** show the combined relationship between maternal thyroid hormones and male newborn birth outcomes; **(G–I)** shows the combined relationship between maternal thyroid hormones and the birth outcomes of female newborns.

We employed Bayesian kernel machine regression (BKMR) to evaluate the combined effects of five maternal thyroid hormones (hereafter referred to as the thyroid hormone mixture) on neonatal birth outcomes. In the first trimester, unstratified analysis revealed positive associations of the thyroid hormone mixture with neonatal birth weight, length, and body parity. After sex stratification, the mixture exhibited weak associations with male birth weight, a weak positive association with male birth length, and a negative association with male parity. For females, weak positive associations were observed between the mixture and birth weight, length, and parity (**Figure 3**). In the second trimester, unstratified BKMR showed significant positive associations of the thyroid hormone mixture with birth weight, length, and parity. These associations persisted after gender stratification but were attenuated (**Figure 4**). In the third trimester, both unstratified and sex-stratified analyses revealed significant positive associations between the thyroid hormone mixture and all three birth outcomes (**Figure 5**).

### Weighted analysis of maternal thyroid hormones and neonatal birth outcomes

**Figure 6:**
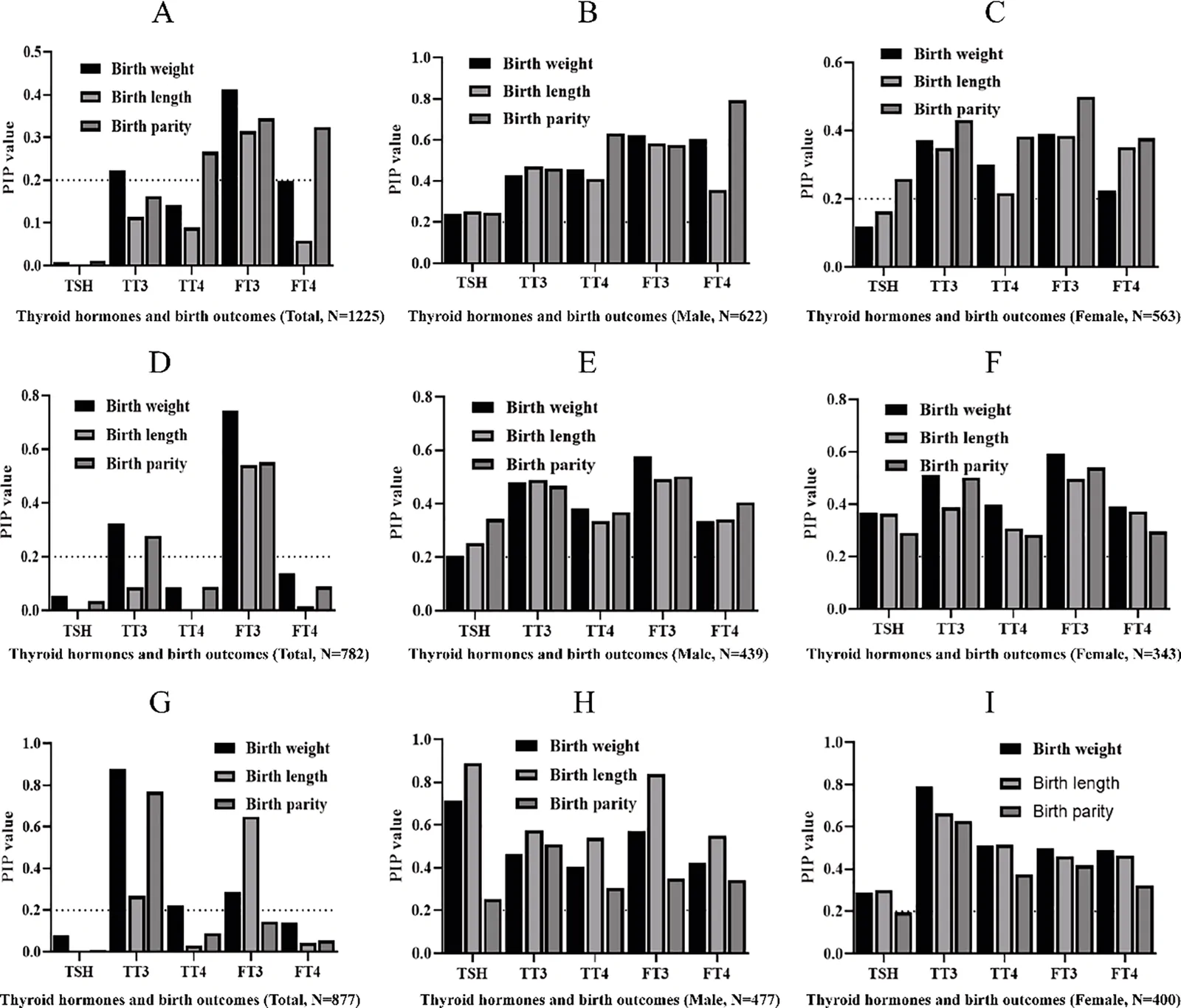
Weighted analysis of maternal thyroid hormones and neonatal birth outcomes. **(A–C)** show the posterior inclusion probabilities (PIP) of maternal thyroid hormones and birth outcomes in the first trimester; **(D–F)** represent the PIP of maternal thyroid hormones and birth outcomes in the second trimester; **(G–I)** shows the PIP of maternal thyroid hormones and birth outcomes in the third trimester. The closer the PIP is to 1, the more likely the variable is to be a key driving factor in the mixture; The closer the PIP is to 0, the less likely or non-critical the variable is to have an impact on the outcome.

To further estimate the relative weight of maternal thyroid hormones at different stages of pregnancy on neonatal birth outcomes, this study used Bayesian kernel machine regression for analysis, with results expressed as posterior inclusion probability (PIP). Results showed that in the first trimester, the contribution weights for newborn birth weight and parity followed the order: FT3 > TSH > TT3 > TT4 > FT4. For birth length, the ranking was: FT3 > TT4 > TSH > TT3 > FT4. In the second trimester, the weight order for birth weight and length was: FT3 > TSH > TT3 > TT4 > FT4, while for birth parity, it was: FT3 > TT3 > TSH > TT4 > FT4. In the third trimester, the hierarchy for birth weight was: TSH > FT3 > TT4 > TT3 > FT4. For birth length, the weights were: FT3 > TSH > TT3 > TT4 > FT4, and for birth parity the weights were: TT4 > TSH > TT3 > FT3 > FT4 (**Figure 6**).

## Discussion

In this study, maternal thyroid hormone levels were lower than those reported elsewhere. For example, one study documented early-pregnancy TT4 levels of 0.11–3.67 mIU/L, FT3 of 3.19–5.91 pmol/L, and FT4 of 10.95–16.79 pmol/L (12). A birth cohort study found early-pregnancy thyroid-stimulating hormone (TSH) of 0.74–5.46 μIU/mL, FT3 of 3.82–5.34 pmol/L, and FT4 of 12.94–20.58 pmol/L (13). Notably, we observed significant pregnancy-stage differences in TSH, TT3, FT3, and FT4. TSH levels increased progressively from early to late pregnancy; TT3 rose from early to mid-pregnancy before declining; FT3 and FT4 decreased continuously from early to late pregnancy. This aligns with prior findings: TT4 and FT4 rise mildly in early pregnancy, followed by a gradual FT4 decline persisting until delivery; TSH decreases in early pregnancy, then increases in mid-late pregnancy while remaining low (5), with thyroid hormone reference ranges varying by gestational stage (6). These dynamics underscore the dynamic nature of maternal thyroid hormone levels during pregnancy.

We observed associations between maternal thyroid hormone levels during pregnancy and neonatal birth weight, length, and body mass index. Previous studies have reported links between maternal thyroid hormones and birth weight (22), with pregnancy TSH negatively correlated with birth weight (5, 14) and elevated FT4 also associated with reduced birth weight (1, 8). In a cohort of 3,988 pregnant women, maternal FT4 showed a sex-specific negative correlation with birth weight (9). Thyroid dysfunction, including clinical or subclinical hypothyroidism, increases low birth weight risk (3), though some studies report no significant associations (4, 11), and others find positive late-pregnancy FT3–birth weight correlations (5). Such heterogeneity likely reflects population differences (race, region, environment) and study design/sample size biases. Notably, we identified dose-response relationships between maternal thyroid hormones and neonatal birth weight, length, and body mass index. This aligns with prior findings: each 1 pmol/L early-pregnancy FT4 increase was associated with 35–50 g lower birth weight (15), and maternal thyroid hormones indirectly regulate birth weight via environmental pollutant interactions (16). A meta-analysis also reported negative dose-response associations between maternal TSH/FT4 (even within normal ranges) and birth weight (1).

Our analyses revealed sex-dependent relationships between maternal thyroid hormones and neonatal birth outcomes, likely arising from inherent sexual dimorphism in fetal thyroid sensitivity, placental function, and epigenetic regulation. Male fetuses show higher thyroid hormone receptor (TRα/TRβ) expression in placental endothelium and fetal liver, increasing susceptibility to FT4-driven reductions in placental perfusion and hepatic IGF-1 expression, and thereby worsening fetal growth restriction (17). Sex-specific placental endocrine profiles—including differential hCG and PLGF secretion—modulate trophoblast proliferation and nutrient transfer under thyroidal control, with female placentas displaying greater adaptive capacity (18). Differences in epiphyseal chondrocyte maturation further drive sex-dependent responses to FT4-mediated skeletal development and birth length outcomes (10). Reciprocally, fetal thyroid maturation influences maternal thyroid status: autonomous hormone synthesis from mid-gestation establishes bidirectional endocrine crosstalk (19). Disrupted maternal FT4 or TSH may impair placental thyroid hormone transfer, delay fetal HPT axis maturation, and alter metabolic and developmental programming (18). Given the tight physiological coupling between maternal endocrine milieu and fetal thyroid development, even subtle maternal thyroid perturbations can exert persistent effects on offspring thyroid function (20).

### Strengths and limitations

This study offers several key advantages. First, it provides a systematic analysis of maternal thyroid hormone dynamics across all gestational trimesters. Second, it employs both single-exposure and combined-exposure models to characterize associations between maternal thyroid hormones and neonatal birth outcomes. Third, it uses the RCS method to evaluate dose-response relationships between maternal thyroid hormones and neonatal outcomes. Finally, it quantifies the relative contribution of individual thyroid hormones to neonatal birth outcomes. Notably, the study has limitations. First, unmeasured confounders—such as maternal psychological and physiological factors that may influence offspring birth outcomes—cannot be fully excluded. Future research should explore the mechanistic links between maternal thyroid hormones and neonatal birth outcomes through experimental and molecular studies.

## Conclusion

This study found that there are dynamic changes in maternal thyroid hormones during pregnancy, and maternal thyroid hormones are associated with neonatal weight, birth length, and birth parity, mainly TSH, TT3, FT3, and FT4. Secondly, this association is stronger in the third trimester than in the first trimester and the second trimester, and the association is stronger in females than in males.

## Data Availability

The raw data supporting the conclusions of this article will be made available by the authors, without undue reservation.
