# Supplementary material for: Associations between maternal thyroid hormones and neonatal outcomes during pregnancy
Source: Front Endocrinol (Lausanne). 2026 Jun 8;17:1850925. doi: 10.3389/fendo.2026.1850925 (PMC13283886; doi:10.3389/fendo.2026.1850925)
Supplement: Supplementary file 1 [file SupplementaryFile1.docx]

| ZBC(**N=6583**)  Complete survey data **(N=6359)**：including maternal age, race, education, occupation, marital status, pre-pregnancy body mass index, smoking history, number of pregnancies, spousal education, spousal occupation, annual household income, and spousal smoking history.  Maternal thyroid hormones **(N=3359)**: Thyroid-stimulating hormone (TSH), total thyroxine (TT4), free thyroxine (FT4), total triiodothyronine (TT3), and free triiodothyronine (FT3)  Neonatal outcomes **(N=2884)**： Birth weight (*g*), birth length (*cm*), birth parity (*kg/m^3^*).  Study population **(N=2884**): 1225 in early pregnancy, 782 in mid-pregnancy, and 877 in late pregnancy |
| --- |
| Figure S1 Study population |

| 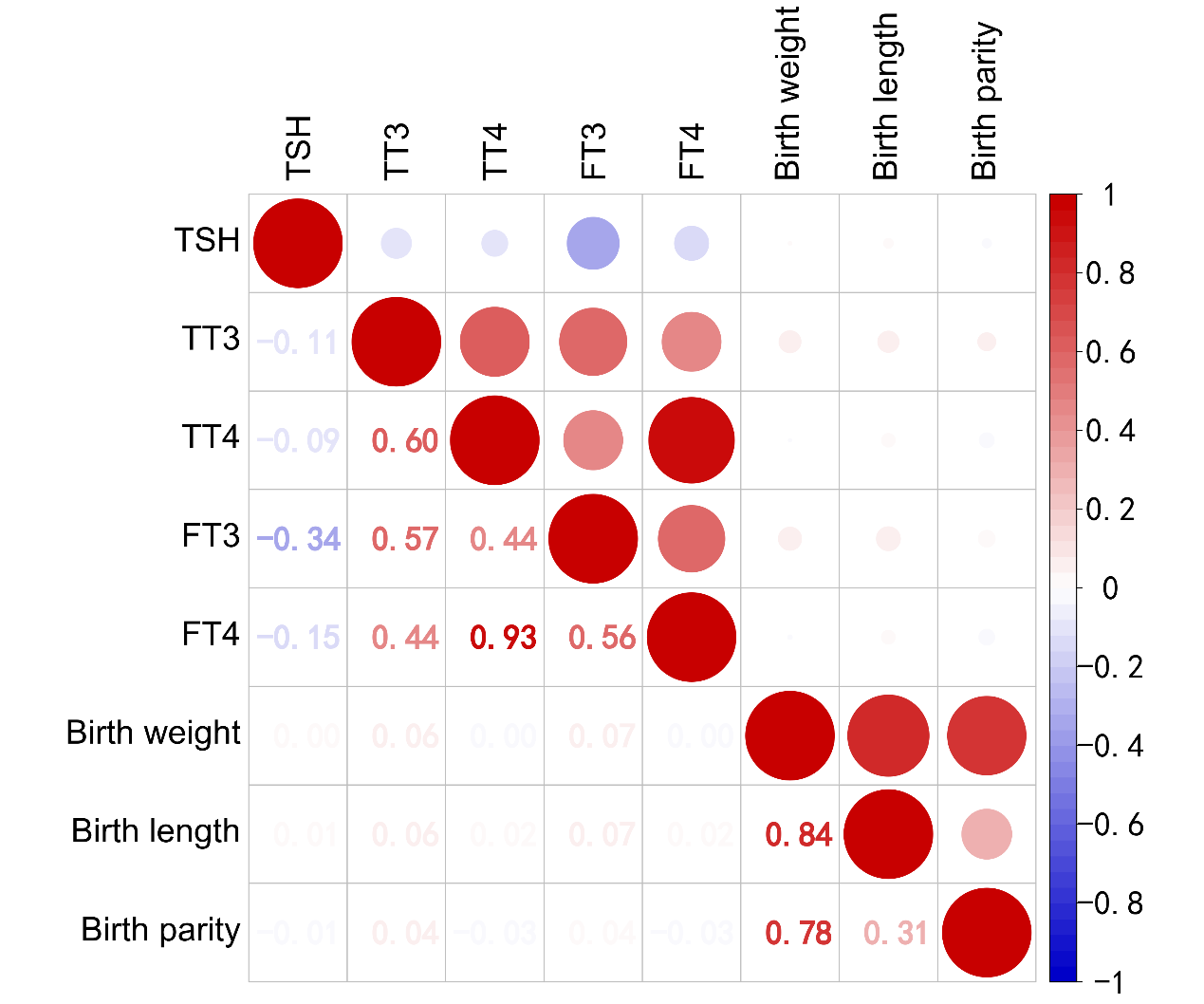 |
| --- |
| Figure S2 Correlation analysis between thyroid hormones and birth outcomes |
